# Trends in the Epidemiology of Candidemia in Intensive Care Units From 2006 to 2017: Results From the Korean National Healthcare-Associated Infections Surveillance System

**DOI:** 10.3389/fmed.2020.606976

**Published:** 2020-12-17

**Authors:** Eun Jin Kim, Eunyoung Lee, Yee Gyung Kwak, Hyeon Mi Yoo, Ji Youn Choi, Sung Ran Kim, Myoung Jin Shin, So-Yeon Yoo, Nan-Hyoung Cho, Young Hwa Choi

**Affiliations:** ^1^Department of Infectious Diseases, Ajou University School of Medicine, Suwon, South Korea; ^2^Department of Biomedical Informatics, Ajou University School of Medicine, Suwon, South Korea; ^3^Office of Biostatistics, Medical Research Collaborating Center, Ajou Research Institute for Innovative Medicine, Ajou University Medical Center, Suwon, South Korea; ^4^Department of Internal Medicine, Inje University Ilsan Paik Hospital, Goyang, South Korea; ^5^Infection Control Office, Inje University Sanggye Paik Hospital, Seoul, South Korea; ^6^Infection Control Unit, Chung-Ang University Healthcare System, Seoul, South Korea; ^7^Infection Control Office, Korea University Guro Hospital, Seoul, South Korea; ^8^Infection Control Office, Seoul National University Bundang Hospital, Seongnam, South Korea; ^9^Adjunct Assistant Professor, College of Nursing, The Catholic University of Korea, Seoul, South Korea; ^10^Department of Infection Control, Gangnam Severance Hospital, Yonsei University, Seoul, South Korea

**Keywords:** bloodstream infections, Candidemia, surveillance, Republic of Korea, intensive care units'

## Abstract

Candidemia is an important healthcare-associated infection (HAI) in intensive care units (ICUs). However, limited research has been conducted on candidemia in the Republic of Korea. We aimed to analyze the secular trends in the incidence and distribution of candidemia in ICUs over 12-years using data from the Korean National Healthcare-Associated Infections Surveillance System (KONIS). KONIS was established in 2006 and has performed prospective surveillance of HAIs including bloodstream infections (BSIs) in ICUs. We evaluated the trends in the distribution of causative pathogens and the incidence of candidemia. From 2006 to 2017, 2,248 candidemia cases occurred in 9,184,264 patient-days (PDs). The pooled mean incidence rates of candidemia significantly decreased from 3.05 cases/10,000 PDs in 2006 to 2.5 cases/10,000 PDs in 2017 (*P* = 0.001). Nevertheless, the proportion of candidemia gradually increased from 15.2% in 2006 to 16.6% in 2017 (*P* = 0.001). The most frequent causative pathogen of BSIs from 2006 to 2012 was *Staphylococcus aureus*; however, *Candida* spp. emerged as the most frequent causative pathogen since 2013. *C. albicans* (39.9%) was the most common among *Candida* spp. causing BSIs, followed by *Candida tropicalis* (20.2%) and *Candida parapsilosis* (18.2%). The proportion of candidemia caused by *C. glabrata* significantly increased from 8.9% in 2006 to 17.9% in 2017 (*P* < 0.001). There was no significant change in the distribution of *Candida* spp. by year (*P* = 0.285). The most common source of BSIs was central lines associated BSI (92.5%). There was a significant increase in the proportion of candidemia by year in hospitals with organ transplant wards (from 18.9% in 2006 to 21.1% in 2017, *P* = 0.003), hospitals with <500 beds (from 2.7% in 2006 to 13.6% in 2017, *P* < 0.001), and surgical ICUs (from 16.2% in 2006 to 21.7% in 2017, *P* = 0.003). The proportion of candidemia has increased in Korea, especially in hospitals with <500 beds and surgical ICUs. Thus, appropriate infection control programs are needed.

## Introduction

Candidemia is an increasingly important healthcare-associated fungal infection associated with high morbidity and mortality (1–3). In addition, it increases the financial healthcare burden and prolongs hospital stay (4). Candidemia is the fifth cause of healthcare-associated bloodstream infections (BSI) in intensive care units (ICUs) in European countries, and in several US states, *Candida* species have been reported as the most prevalent pathogens causing healthcare-associated BSIs (5, 6). In particular, increasing incidence rates have been observed in ICUs, particularly among immunocompromised patients, patients treated with broad-spectrum antibiotics, and patients requiring invasive procedures and devices (7). The epidemiology of candidemia varies according to the geographical region, period, type of survey, and the population involved (8–10). Recent epidemiological studies have reported an increasing incidence of non-albicans candidemia. *Candida albicans* is still the predominant species; however, *Candida glabrata*, which is less susceptible to antifungal drugs, ranks second in the USA, Northern Europe, and Australia, while *Candida parapsilosis* is the most prevalent non-albicans species in Latin America, Southern Europe, and Asia (8, 9, 11). Therefore, research on the distribution of *Candida* spp. and the incidence of candidemia in each country is important for optimizing prevention and treatment strategies. Limited research has been conducted on candidemia in the Republic of Korea.

The Korean National Healthcare-Associated Infections Surveillance System (KONIS) is a nationwide network for the prospective surveillance of healthcare-associated infections (HAIs), including the surveillance of causative pathogens. It was established in 2006 by the Korea Center for Disease Control and Prevention and the Korean Society for Healthcare-Associated Infection Control with the aim of improving infection control practices in hospital ICUs (12). This study aimed to describe the impact of candidemia by analyzing the trends in the incidence and proportion of candidemia using national cohort data from the KONIS. We also aimed to identify secular trends in candidemia according to the characteristics of hospitals and ICUs to provide information for assisting the development of infection control policies.

## Materials and Methods

In the Republic of Korea, the KONIS continuously monitors HAIs and causative pathogens, including BSIs in the ICU. This system focuses on the surveillance and prevention of HAIs, including device-associated infections, in adult patients in ICUs. KONIS surveillance was conducted in participating hospitals from 2006 to 2017. Hospital participation was voluntary, and the results are handled confidentially. The definitions of HAIs and device utilization ratio (DUR) were standardized and based on those of the CDC/NHSN system (13). In total, 285 ICUs and 217 hospitals with over 200 beds participated. These hospitals represented more than 70% of the hospitals with over 200 beds in the Republic of Korea.

In this study, we only considered patients older than 15 years who developed candidemia during their stay in the ICU. All patients who stayed in the ICU for >2 days were included in the surveillance and were followed-up from admission until discharge or death. After ICU discharge, patients were followed-up for infection for an additional 2 days. Candidemia was defined as at least one positive blood culture for *Candida* spp. in ICU patients hospitalized for more than 48 h. For patients with multiple episodes of candidemia within a 14-day period, only the first episode was included. Detection and identification of *Candida* spp. were performed in the notifying laboratories according to standard protocols in use in each facility. A central line-associated BSI (CLABSI) was defined as a primary BSI in a patient with a central line within the 48 h preceding the development of the BSI that was not bloodstream related to an infection at another site. Annual incidence rates of all HAIs including BSI were calculated as the number of infections per 1,000 patient-days (PDs) for each year of participation. The pooled incidence rates of device-associated HAIs and DUR were calculated for each year. The annual incidence rates of BSIs caused by each pathogen were calculated as the number of BSIs per 10,000 PDs to enable comparison with other data and increase readability. The annual percentages of gram-positive, gram-negative, and fungal pathogens causing BSIs were determined for trend analysis. Trends in the proportion of candidemia were analyzed for *C. albicans* candidemia and non-albicans (*C. glabrata, C. tropicalis*, and *C. parapsilosis*) candidemia. We also collected data on the organizational and institutional characteristics of the included hospitals and ICUs, as described in a previous report (14).

### Statistical Analyses

We evaluated secular trends in the annual incidence and proportion of the causative pathogens and candidemia. Trends in incidence rate estimates were tested using a negative binomial regression model with the year as a linear predictor. The Cochran–Armitage test and Cochran–Mantel–Haenszel mean score test were performed to identify trends of proportions after adjusting for organizational and institutional characteristics of the hospitals and ICUs, such as the total number of hospital beds, type of ICU, and whether the presence of a transplant ward or whether the infection was central line-related. All statistical analyses were performed using SAS software (Version 9.4, SAS Institute, Cary, NC, USA). A *P* < 0.05 was considered to be statistically significant.

### Ethical Declaration

This study used information that was disclosed to the general public as national data and was exempted from institutional review board approval because it did not collect or record personally identifiable information.

## Results

**Figure 1:**
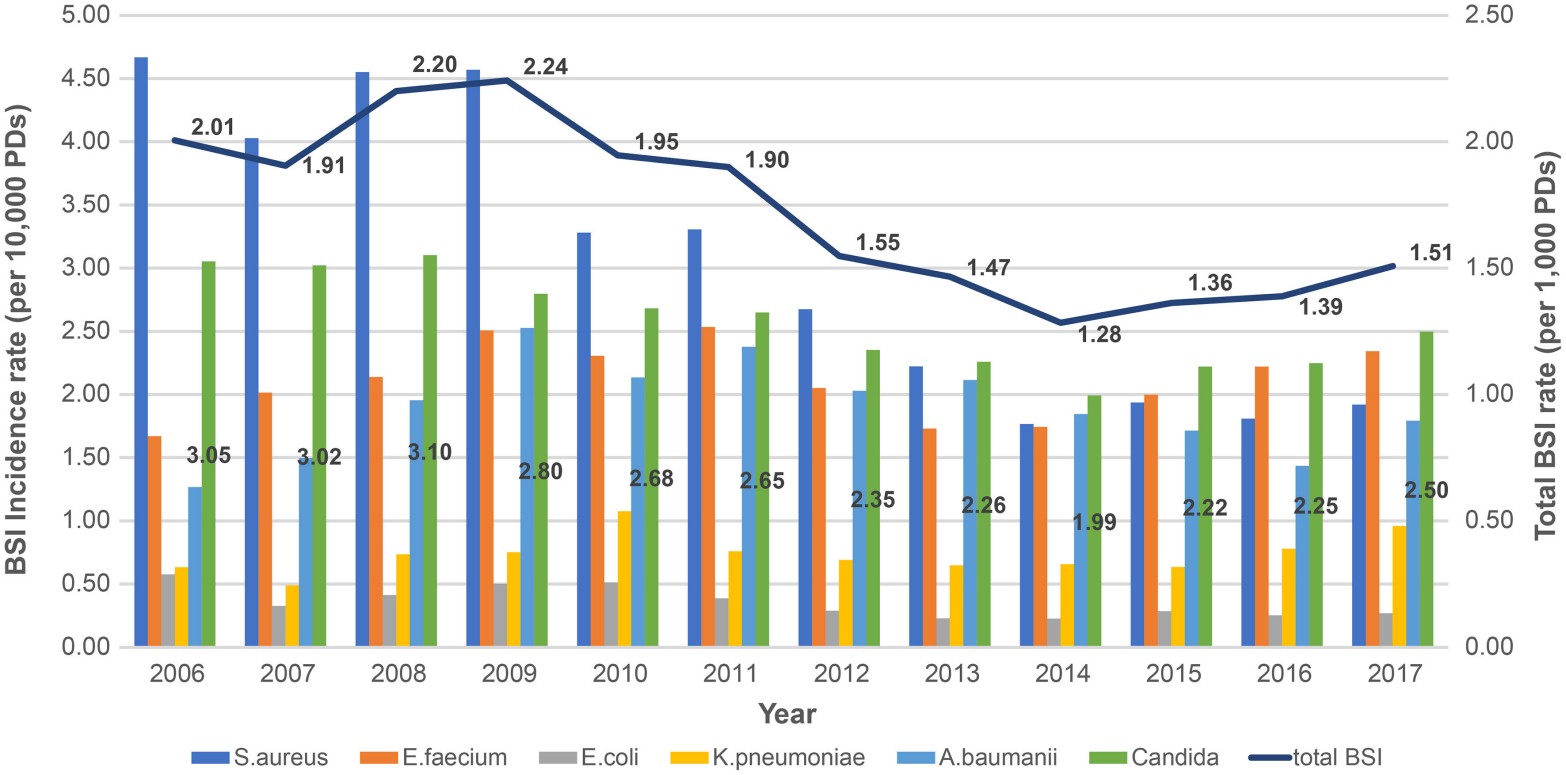
Changing yearly trends in total bloodstream infection incidence rates and pathogen-specific bloodstream infection incidence rates during 2006–2017 based on data from KONIS. BSI, bloodstream infection; PDs, patient-days; KONIS, Korean National Healthcare-Associated Infections Surveillance System.

**Figure 2:**
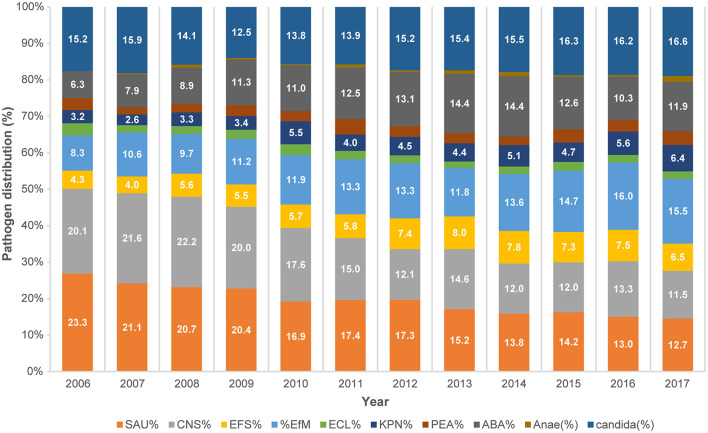
Trends in the distribution of pathogens causing bloodstream infections during 2006–2017 based on data from KONIS. SAU, *Staphylococcus aureus*; CNS, coagulase-negative *Staphylococci*; EFS, *Enterococcus faecalis*; EFM, *Enterococcus faecium*; ECL, *Escherichia coli*; KPN, *Klebsiella pneumoniae*; PEA, *Pseudomonas aeruginosa*; ABA, *Acinetobacter baumannii*; Anae, anaerobic pathogen; KONIS, Korean National Healthcare-Associated Infections Surveillance System.

The pooled mean BSI incidence rate decreased since the initiation of the survey from 2.01/1,000 PDs in 2006 to 1.51/1,000 PDs in 2017 (*P* < 0.001, Figure 1). Overall, 2,248 candidemia cases occurred in 9,184,264 PDs. The total pooled mean incidence rate was 2.4 cases/10,000 PDs during 2006–2017, and the majority of infections were CLABSIs (92.5%). The pooled mean incidence rates of candidemia decreased from 3.05 cases/10,000 PDs in 2006 to 2.5 cases/10,000 PDs in 2017 (*P* = 0.001). From 2006 to 2012, *Staphylococcus aureus* was the most frequently identified pathogen. However, *Candida* spp. emerged as the most frequently identified pathogen from 2013 to 2017 (Figure 1). Furthermore, the proportion of BSIs caused by candidemia significantly increased from 15.2% in 2006 to 16.6% in 2017 (*P* = 0.001, Figure 2).

**Figure 3:**
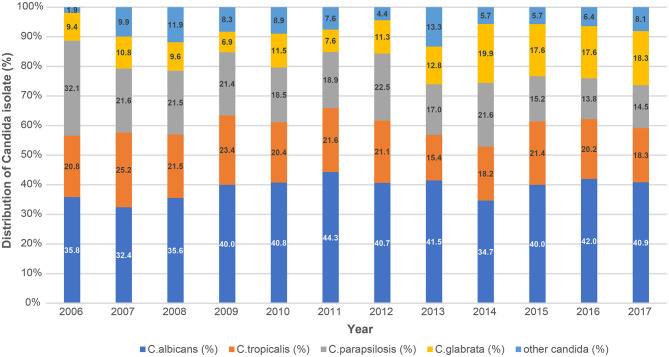
Trends in the distribution of *Candida* species causing bloodstream infections during 2006–2017 based on KONIS. KONIS, Korean National Healthcare-Associated Infections Surveillance System.

Among the cases of candidemia, *C. albicans* (*n* = 896, 39.9%) were the most frequently identified pathogen, followed by *C. tropicalis* (*n* = 454, 20.2%) and *C. parapsilosis* (*n* = 410, 18.2%). There were no significant changes in the proportions of *C. albicans* and non-albicans candidemia by year (*P* = 0.285). *C. glabrata* (*n* = 314, 14.0%) did not have a high incidence rate, however, the proportion of candidemia caused by *C. glabrata* increased considerably from 8.9% in 2006 to 17.9% in 2017 (*P* < 0.001). In contrast, the proportion of candidemia caused by *C. parapsilosis* substantially decreased (*P* < 0.001); no significant trend was observed in the proportion of candidemia caused by *C. tropicalis* (*P* = 0.151) (Table 1, Figure 3).

**Table 1:** Distribution of *Candida* species during 2006–2017 based on data from the Korean National Healthcare-Associated Infections Surveillance System (KONIS).

**Year**	**Participants hospital, *n***	**Total patient-days**	***C. albicans n*, (%)**	***C. parapsilosis n*, (%)**	***C. tropicalis n*, (%)**	***C. glabrata n*, (%)**	**Other *n*, (%)**	**Total *n*, (%)**
2006	44	173,559	19 (35.8)	17 (32.1)	11 (20.8)	5 (9.4)	1 (1.9)	53 (100)
2007	56	367,352	36 (32.4)	24 (21.6)	28 (25.2)	12 (10.8)	11 (9.9)	111 (100)
2008	57	435,035	48 (35.6)	29 (21.5)	29 (21.5)	13 (9.6)	16 (11.9)	135 (100)
2009	63	518,620	58 (40.0)	31 (21.4)	34 (23.4)	10 (6.9)	12 (8.3)	145 (100)
2010	72	585,325	64 (40.8)	29 (18.5)	32 (20.4)	18 (11.5)	14 (8.9)	157 (100)
2011	81	698,595	82 (44.3)	35 (18.9)	40 (21.6)	14 (7.6)	14 (7.6)	185 (100)
2012	91	867,683	83 (40.7)	46 (22.5)	43 (21.1)	23 (11.3)	9 (4.4)	204 (100)
2013	94	832,428	78 (41.5)	32 (17.0)	29 (15.4)	24 (12.8)	25 (13.3)	188 (100)
2014	96	883,138	61 (34.7)	38 (21.6)	32 (18.2)	35 (19.9)	10 (5.7)	176 (100)
2015	103	945,605	84 (40.0)	32 (15.2)	45 (21.4)	37 (17.6)	12 (5.7)	210 (100)
2016	193	1,387,515	131 (42.0)	43 (13.8)	63 (20.2)	55 (17.6)	20 (6.4)	312 (100)
2017	216	1,489,409	152 (40.9)	54 (14.5)	68 (18.3)	68 (18.3)	30 (8.1)	372 (100)
2006–2017	216	9,184,264	896 (39.9)	410 (18.2)	454 (20.2)	314 (14.0)	174 (7.7)	2248 (100)

We performed subgroup analysis to identify the hospital characteristics associated with a high incidence of candidemia (Table 2). In the subgroup analysis, the increase in the proportion of candidemia by year was significantly higher in hospitals with <500 beds (from 2.7% in 2006 to 13.6% in 2017, *P* < 0.001), those with surgical ICUs (from 16.2% in 2006 to 21.7% in 2017, *P* < 0.001), and hospitals with organ transplant wards (from 18.9% in 2006 to 21.1% in 2017, *P* = 0.003). The proportion of CLABSIs did not differ significantly by year (*P* = 0.169).

**Table 2:** Proportion of candidemia in ICUs during 2006–2017 with subgroup analysis according to hospital or pathogen characteristics.

**Subgroup**	**Year**												***P*-value**
	**2006**	**2007**	**2008**	**2009**	**2010**	**2011**	**2012**	**2013**	**2014**	**2015**	**2016**	**2017**	
Candidemia (%)	15.23	15.86	14.11	12.47	13.78	13.94	15.19	15.41	15.52	16.3	16.2	16.56	<0.05^a^
CLABSI, yes (%)	93.24	92.79	93.23	94.08	88.96	93	94.86	94.97	92.41	91.78	91.33	91.44	0.17^a^
*Candida* species (%)													0.29^b^
*C. albicans*	31.08	32.43	36.09	38.16	39.26	42.5	38.79	39.20	34.18	38.36	40.87	40.37	
Non-*albicans* spp.	66.22	66.67	63.91	55.92	56.44	52	57.48	55.28	62.66	57.53	56.35	58.02	
Non-*Candida* fungi (%)	2.7	0.9	0	5.92	4.29	5.5	3.74	5.53	3.16	4.11	2.79	1.6	
Size of hospitals (%)													<0.05^b^
300–699 beds	2.7	0.9	5.26	1.97	4.91	2.5	5.14	8.04	6.96	6.39	11.76	13.64	
700–899 beds	51.35	48.65	60.15	48.68	47.85	58.5	61.68	53.77	48.1	58.45	56.97	58.02	
≥900 beds	45.95	50.45	34.59	49.34	47.24	39	33.18	38.19	44.94	35.16	31.27	28.34	
Type of ICU (%)													<0.05^b^
Medical ICU	83.78	83.79	78.19	77.63	81.59	75.5	67.76	72.86	68.98	71.24	76.16	78.34	
Surgical ICU	16.22	16.22	21.8	22.37	18.4	24.5	32.24	27.14	31.01	28.77	23.84	21.66	
Organ transplant wards, yes (%)	18.92	31.53	33.83	32.89	29.45	35.00	27.57	30.15	31.65	25.57	21.98	21.12	<0.05^a^

*The P-value was obtained using the one-sided ^a^Cochran-Armitage trend test or ^b^the one-sided Cochran-Mantel-Haenszel mean score test*.

*CLABSI, central line-associated bloodstream infection; ICU, intensive care unit*.

## Discussion

Invasive candidiasis has emerged as an important public health problem associated with a high mortality rate. The most common presentation of invasive candidiasis is candidemia. Candidemia frequently occurs in ICUs, even in non-immunocompromised patients, and increases the length and costs of ICU stay. *Candida* spp. are the fourth most common causative agents of healthcare-associated BSIs in the USA, accounting for 8–10% of all BSIs acquired in the hospital (4, 7, 15). The global incidence rate of candidemia is increasing due to an increase in the population receiving immunosuppressive therapy, a growing elderly population, and an increase in the survival of patients with previously considered lethal diseases (3, 11, 15). Various studies have shown that the incidence rate of candidemia in ICU settings ranges from 0.7 to 23.1 cases per 10,000 PDs (16, 17).

In this report, we estimated the pooled mean incidence rate of candidemia to be 2.4 cases/10,000 PDs, and the annual incidence rate was found to decrease from 3.05 cases/10,000 PDs in 2006 to 2.5 cases/10,000 PDs in 2017. In another study in the Republic of Korea, the average incidence of candidemia at nine university hospitals in 2011 was 2.01 cases/10,000 PDs (18). The participating hospitals' overall HAI incidence rate was found to have decreased significantly during the study period (14). The CLABSI incidence rate showed a particularly marked decrease since continuous KONIS surveillance (19). We believe that participation in continuous national surveillance has contributed to a significant reduction in BSI and candidemia incidence rates.

But noteworthy, a significant increase has been observed in the proportion of BSIs involving candidemia, *Candida* spp. have been the most commonly encountered BSI pathogens since 2013 in this study. A point-prevalence study conducted in several US states found that *Candida* spp. were the most prevalent healthcare-associated BSI pathogens; their prevalence exceeded those of some common bacterial species (5). This surprising result that *Candida* spp. is the most common pathogen of BSI has significant implications, such as increased incidence rate observed in the other studies mentioned previously. This may require more accurate, and early treatment and a new challenge for infection control. This may be due to a combination of factors such as an increase in elderly patients, an increase in complex ICU patients, an increase in invasive procedures, more extensive surgical procedures, and an increase in antibiotics use including antifungal agents (20).

As is well known, ICU patients have high candidemia risk than the general ward; higher candidemia rates were reported. A retrospective study conducted in Belgium showed a significant increase in candidemia incidence from 0.86 cases/10,000 PDs in 2006 to 2.12 cases/10,000 PDs in 2012, driven by ICUs. The incidence of candidemia in ICUs increased to 10.7/10,000 PDs, whereas candidemia incidence in the non-ICU wards decreased (2). A meta-analysis of population-based studies in Europe (3) showed an increase in the overall pooled incidence rate over 10 years, and the highest pooled incidence rate was observed in ICUs (5.5/1,000 admissions). The increased proportion of candidemia seen in this study was more pronounced because it was ICU surveillance. Our challenge is that these ICU patients not only have a higher incidence rate but have also proven to have a higher mortality rate that is affected by the underlying diseases (21, 22). Therefore, it is important to prevent and manage candidemia in ICU settings, and it is necessary to conduct continuous surveillance for candidemia as a key indicator of infection control. Early intervention strategies that facilitate identifying of high-risk candidates to receive early antifungal treatment were needed to improve ICU patients with candidemia outcomes (23). According to the IDSA recommendations, a safe and effective prophylactic strategy, including appropriate antifungal use to prevent candidemia among high-risk patients and daily bathing with chlorhexidine could be considered (24). Infectious disease consultation is one of the best-proven ways to lower patients' mortality rates with candidemia (25).

The high proportion of candidemia found in this study may be attributable to the use of more invasive devices. In a previous KONIS study, the DUR was reported to remain similar over 10-years without a significant decrease in the DUR for C-lines (14). A significant increase in the proportion of candidemia occurred in hospitals with <500 beds. The central line DUR increased slightly in hospitals with 500 beds or less, although this increase was not significant. The lack of reduction in DUR is likely to have contributed to the high proportion of candidemia. In a survey of participating hospitals, the proportion of appropriate use of sterile full body drape and antiseptics during C-line insertion was not enough (55–72%, respectively) (26). Therefore, the proper application of the C-line insertion practice and reducing the DUR may reduce candidemia (27). Appropriate training and adherence monitoring for C-line insertion practice and maintenance will continue to be needed.

The increase in the proportion of candidemia found in this study may be attributable to increasing accuracy of identification tests, such as matrix-assisted laser desorption ionization time-of-flight mass spectrometry, and increasing use of broad-spectrum antibiotics including antifungal agents (28). Fluconazole use has increased by ~4-fold in the past decade (20). The antibiotic stewardship program and monitoring of antimicrobial consumption may be helpful, therefore, its consideration is needed (29).

It is well known that surgical patients especially abdominal operations have a high risk of candidemia. It could have damaged the gastrointestinal barriers and caused skin colonization at vascular insertion sites, which placed these patients at greater risk for developing invasive candidiasis (23), as observed in this study. The increase in the proportion of candidemia in smaller hospitals with <500 beds might be caused by inadequate infection prevention and control support (30). Appropriate infection prevention and control programs such as central line removal and early antifungal therapy may be helpful (31).

There was a substantial reduction in the incidence of BSIs caused by *S. aureus*. A recent study from USA also reported that the overall incidence of *S. aureus* infections had declined slightly (32). Other domestic studies have also reported that the incidence density of ICU-acquired MRSA bacteremia decreased by 20% annually (33). Improved infection control using chlorhexidine, hand hygiene, and reductions in the length of hospital stay have been reported to be associated with a decreased incidence of infections caused by hospital-associated epidemic strains in time-series analyses (34). The reduction in the most common pathogen may have contributed to an increase in the candidemia proportion. However, an increase in candidemia proportion cannot reduce its importance. With the increase in the proportion of HAIs involving candidemia, it is important to implement infection control measures directed at fungal pathogens differently from bacteria by analyzing of risk factors.

Human diseases are caused by at least 15 distinct *Candida* spp.; more than 90% of invasive candidiases are caused by the five most common pathogens: *C. albicans, C. glabrata, C. tropicalis, C. parapsilosis*, and *C. krusei* (24). Although *C. albicans* remains the most prevalent species, there has been a shift in prevalence toward non-albicans *Candida* spp. in recent decades, especially in ICU patients. The epidemiology of candidemia differs by country. *C. albicans* was the predominant species in most studies (range 37.9–76.3%). *C. glabrata* was the second most commonly isolated *Candida* spp. in North America, North China, and North Europe. In contrast *C. parapsilosis* has emerged in Southern Europe and Latin America as a causative agent of non-albicans candidemia (2, 17, 35). In this study, no significant shift was observed toward non-albicans *Candida* spp., and *C. albicans* remained the dominant cause of candidemia. In a multicenter study conducted in the Republic of Korea, the distribution of *Candida* spp. was as follows: *C. albicans* (38–40.4%), *C. parapsilosis* (19.9–26%), *C. tropicalis* (17.6–20%), *C. glabrata* (11–14.2%), and miscellaneous *Candida* spp. (5%), which is similar to the distribution found in this study (18, 36). However, this study's important finding was the significant increase in the annual proportion of *C. glabrata* candidemia. The proportion of *C. glabrata* candidemia has been increasing worldwide (8, 9, 11, 37). In a retrospective epidemiological study of candidemia in Japan, *C. parapsilosis* was the second most common species, but the proportion of *C. glabrata* was significantly increased (38). In this study, the decreased incidence of *C. parapsilosis* candidemia offset the increase in the incidence of *C. glabrata* candidemia; thus, there was no significant increase in the overall proportion of non-albicans candidemia. Since *C. parapsilosis* colonizes the skin, it is a common cause of catheter-related infections. In contrast, *C. glabrata* candidemia is more common among older patients and patients with malignancies (17). Therefore, the increasing proportion of *C. glabrata* candidemia may reflect the increase in the burden of immunocompromised and elderly patients in ICUs. This report also revealed a significant increase in the proportion of candidemia among patients in organ transplant wards. The increase in *C. glabrata* can be interpreted in association with this. In addition, increasing the use of antifungal and broad-spectrum antibiotics may have contributed. Fluconazole prophylaxis was the single most important determinant (20, 39).

*C. glabrata* has been reported to show antifungal resistance, including an intrinsically decreased susceptibility to azole agents and resistance to amphotericin B (37). BSIs caused by non-albicans *Candida* spp. are difficult to treat due to antifungal resistance and have a high mortality rate (40). According to an epidemiologic study conducted in Europe, mortality was higher in patients with non-albicans *Candida* infections than those with *C. albicans* infection (47.3 vs. 32.4%, respectively) (3, 35). Thus, continuous monitoring and research on precipitating factors for the increasing proportion of *C. glabrata* candidemia shown in this study are important. Additionally, an antifungal stewardship program is needed.

*C. auris*, a problematic strain, has no reports of outbreaks in healthcare settings in Republic of Korea, and there was no such case in this study (41, 42).

The incidence of non-albicans candidemia has been reported to be high among patients receiving total parenteral nutrition (TPN), while *C. albicans* candidemia has been more common among surgical patients (35). This study also found a significant increase in the proportion of candidemia in surgical ICUs. Although we did not collect data on TPN, our findings highlight the importance of infection control in surgical ICUs.

The present study had several limitations. We did not include results on isolate susceptibility and changes in antifungal consumption. A previous study conducted in the Republic of Korea found a low resistance rate to fluconazole (1.4%, 9/636) in *Candida* isolates (43). The monitoring of antifungal susceptibility should be included in future surveillance. We could not review detailed clinical information of the patients, the severity of their illnesses, and related mortality. Data of community-acquired BSIs were excluded, while the general wards other than the ICU were not included. Nevertheless, this study provides important information on the trends in infection control of candidemia by analyzing the epidemiology and related hospital characteristics over a 12-year period. It also confirms the importance of candidemia control in resource-limited hospitals such as those with <500 beds and surgical ICUs. Continued research is needed to identify interventions associated with these decreasing trends to accelerate further the reduction in incidence, including improved prevention and treatment strategies, such as the optimal use of antifungal treatments and reduction in the unnecessary use of catheters.

## Conclusions

The proportion of candidemia has increased in the Republic of Korea, specifically in hospitals with <500 beds and surgical ICUs. Moreover the proportion of candidemia caused by *C. glabrata* has gradually increased. Therefore, appropriate infection control programs are needed.

## Data Availability Statement

The raw data supporting the conclusions of this article will be made available by the authors, without undue reservation.

## Author Contributions

YC conceived the study, oversaw the drafting of the manuscript, and supervised and mentored EK, who wrote the first draft. SK, MS, HY, JC, and N-HC contributed to data extraction and clearing. YK and S-YY provided specific expertise on epidemiology and contributed to data management. EL contributed to statistical analysis and interpretation of data. All authors critically reviewed successive drafts of the manuscript, provided important intellectual input, and approved the final version for publication.

## Conflict of Interest

The authors declare that the research was conducted in the absence of any commercial or financial relationships that could be construed as a potential conflict of interest.

## Abbreviations

BSI: bloodstream infection
CLABSI: central line-associated bloodstream infection
DUR: device utilization ratios
HAIs: healthcare-associated infections
KONIS: the Korean National Healthcare-Associated Infections Surveillance System
PDs: patient-days.
